# Forceps Delivery during the Last Fifty Years

**Published:** 1892-09

**Authors:** Joseph Griffiths Swayne

**Affiliations:** Consulting Physician-Accoucheur to the Bristol General Hospital, Lecturer on Midwifery at the Bristol Medical School


THE BRISTOL

Medico-Chirurgical Journal.

SEPTEMBER, 1892.

### FORCEPS DELIVERY DURING THE LAST FIFTY YEARS.

BY

JOSEPH GRIFFITHS SWAYNE, M.D. Lond.,

*Consulting Physician-Accoucheur to the Bristol General Hospital, Lecturer on Midwifery at the Bristol Medical School.*

A HALF-CENTURY'S experience in any department of the healing art ought always to be worth recording; but to be of any real value, such a record should not depend solely on a man's memory, which is necessarily fleeting and fallible, but should be founded upon a series of facts, each of which he has carefully noted at the time of its occurrence. Since I first began practice in 1842, I have kept accurate notes of all my obstetric cases; and I therefore venture in 1892 to submit to your notice the experience which I have acquired respecting *Forceps Delivery*. Before 1842, when I was not yet a qualified practitioner, I attended thirty-five midwifery cases under the superintendence of my father, who was then lecturer on obstetrics at the Bristol Medical School; but as I did not at that time keep a proper register of cases so attended, and as (to look at the matter from a student's point of view) I was not "fortunate enough" to meet with a forceps case before 1842, I shall make no further allusion to my experience as a student; nor, in relating my experience as a qualified practitioner, shall I refer to those cases in which I had to use the forceps simply to hasten delivery on account of complications, dangerous either to mother or child, or both, such as convulsions, hemorrhages before labour, prolapse of the cord, &c.

All these cases have special dangers and difficulties of their own, and have no bearing on the use of the forceps in difficult labour *per se*. It is in cases of this latter kind that the forceps is of such inestimable value. In difficult labour it supplements the powers of Nature when deficient; it helps them to overcome mechanical obstacles to the passage of the child, arising either from want of room in the pelvic canal, or unusual size of the child's head or malpresentation of the same. It is necessary to lay this stress upon the head, because it is the only part to which the forceps is applicable. No man in his senses would dream of applying the forceps over the body to assist a tedious case of breech presentation, for instance. From January 1st, 1842, up to January 1st of the present year, I have met with 220 cases of tedious and difficult labour in which it was necessary to have recourse to the forceps. Out of the whole number of 220 cases, I was the original attendant (who had charge of each case from the beginning to the end) in rather more than half, viz. 114, whilst the remainder, viz. 106, were cases to which I was called in to consult with other practitioners who were the original attendants. It is obvious that, if we wish to draw any just conclusions as to the success or otherwise of forceps operations, consultation cases should be strictly excluded.

In the early part of my career ignorant midwives and unskilful practitioners were much more common than they are now, and one was frequently called in to neglected cases that were almost moribund, or, at all events, to unusually bad cases in the hands of more skilful practitioners. It is therefore obvious that we cannot draw any just statistical conclusions from this source, but must base them solely on those cases which were attended from the beginning to the end by the same practitioner.

During the course of this paper it will be convenient to divide the fifty years into five periods of ten years each, noticing, as we pass on, any alterations and improvements in midwifery which have taken place in each decade, and which have a special bearing on the subject of forceps delivery. One of the most striking characteristics of modern midwifery is the more frequent use of the forceps in difficult labour, a change of practice which my own experience, as well as that of others, proves to have been attended with the best results. It was far otherwise, however, during the first decade of my practice, from 1842 to 1852. The forceps was then used very rarely, and with extreme caution, I may almost say timidity. This arose in a great measure from the following cause: During the middle of the last century Smellie, the greatest English accoucheur of that period, greatly improved the forceps, and used it frequently and with much success; the natural result of this was that the instrument rose very highly in favour with the medical profession, and was resorted to much too often, and not seldom with disastrous results. Nor can we wonder at these results when we consider the imperfect state of obstetrical education at that period. Hence a powerful reaction against the instrument arose towards the end of the last century, and was started by such distinguished obstetric physicians as Dr. W. Hunter, Dr. Osborne, and Dr. Denman. All these men powerfully inculcated an implicit reliance on the efforts of Nature, until at last the practice of midwifery became in the highest degree expectant. The effect of this recoil of the pendulum had scarcely passed away, even in the middle of the present century. The following passage, which I quote from Dr. Ramsbotham (whose work was then the favourite text-book), shows very well the prevalent dread of the long forceps: "I must not close my remarks without coinciding in opinion with Professor Davis, that the instrument, although very powerful and valuable, is at the same time very dangerous in its use; that it should not be taken in hand except by those who have acquired some proficiency in operative midwifery; and that it is to be had recourse to, more as an experimental measure for superseding the necessity of destroying the child, than as one of the common resources of our art." [1]

It was under the influence of such doctrines that I first had to perform a forceps operation in 1842. The head was arrested rather high up in the pelvic cavity; and as I could not make the blades of the forceps lock properly, I sent for my father, who soon effected delivery. He explained to me that my failure arose rather from timidity than any other cause. By repressing the head a little with his left hand, he succeeded in reaching the anterior ear and passing the blade of the short forceps

[1] *Obstetric Medicine and Surgery.*

over it. By this it is evident that my father acted in strict observance of Denman's twelfth aphorism on the use of the forceps, which is as follows: "The forceps should always be applied over the ears of the child; it must, therefore, be improper to apply them before an ear can be felt." This is an excellent practical rule; but at that time it was too implicitly obeyed. At the present time, on the contrary, as I shall show by-and-by, it is too much neglected; for it is an undoubted fact that the ear of the child is the most sure and certain guide to the proper application of the forceps. And a proper application of the forceps to the head is more than half the battle in using it with success. And here *in limine* I must beg to differ entirely from those practitioners who, in their text-books, advise that the forceps should be applied solely with relation to the pelvis and without regard to the child's head, and who say that it is no matter how the head is seized so long as it *is* seized. I grant that, in using the *long forceps*, we often act in this way; but, then, we do so from necessity rather than from choice, because the prominent sacrum or other pelvic deformity, which is generally present in such cases, obliges us to introduce the instrument in that part of the pelvis where we can find most room for it. But still, even then it is best, if we can, to apply the forceps so as to grasp the transverse diameter of the head. This, I need hardly say, is the most favourable manner in which we can grasp it; in fact, I believe it to be the only manner which is thoroughly compatible with the safety of both mother and child. When the forceps has been applied in the best way, the blades of the instrument correspond to a line drawn from the vertex to the chin of the child, a diameter a little in front of the longest or occipito-mental diameter, and usually corresponding to the axis of the pelvic cavity. The extremities of the blades ought to reach about half-way between the angles of the jaw and the point of the chin, and the fenestræ should enclose, in their widest part, the ears, and in their narrowest part, near the shanks, the parietal protuberances. The blades will then press on the angles of the jaws, and the malar and frontal bones in front and the mastoid processes behind, all of which are parts well adapted to bear pressure without injury. When, on the contrary, the forceps is applied obliquely—one blade, for instance, resting on the side of the forehead, and the other on the occiput—it is found to be difficult, or almost impossible, to lock the instrument; and if it does not slip off at once, which is most likely, the head is grasped very unevenly, so that one edge of each blade presses deeply into the scalp, or flesh of the face, whilst the other projects from the head, and makes most injurious pressure on the vaginal walls and soft parts of the mother. If, with the instrument so applied, much extractive force or compressing power be applied, the face of the child may be deeply marked, the eyes or the nose much injured, ulceration and sloughing of the integuments and even exfoliation of a portion of the frontal bone produced, as I myself witnessed in one case. In another instance paralysis of the portio dura resulted from the pressure of the point of the instrument upon the nerve behind the jaw. If the blades be applied unevenly, and especially if one be passed too far, and the end project beyond the angle of the jaw, it may compress a loop of cord (if, as is often the case, it be coiled round the neck), and thus cause the death of the child. I have met with several undoubted instances of death thus occasioned. Consequences nearly as disastrous may arise from the pressure of unevenly applied forceps on the soft parts of the mother, such as sloughing of the vagina, crushing of the sacral nerves, and lacerations of the perineum.

As to the blade of the forceps which should be introduced first, this is a matter which depends upon whether or not we can reach the ear. When the head is below the pelvic brim, as in the majority of cases it is, we can generally reach the anterior ear; and there is no question, I think, that the older accoucheurs are right when they recommend that the anterior blade should be first introduced and adjusted to the ear, so as to govern the position of the posterior blade, which is to be introduced after it. There is, however, by no means a general agreement on this point; some authorities, on the contrary, recommending that the posterior blade, being the easiest to introduce, should be passed first. With this recommendation I can by no means concur when the case is one for which the short forceps is suitable; but it is quite a different matter when the head is arrested at the brim, and no ear can be reached. In such a case no instrument but a pair of long forceps with a lateral curve should be used. We have then a more difficult state of things to encounter, and delivery becomes an operation involving much anxiety and requiring much skill. We lose our sure and certain guide, the ear, and have to apply the forceps in a more haphazard way by the guidance of the sutures and fontanelles. Moreover, we have to apply it so that the concavity of its lateral curve is turned towards the front of the pelvis, or the instrument will not follow the curve of the pelvic canal. The forceps will then have to be adjusted rather with relation to the pelvis than to the child's head. Each blade will be situated somewhat obliquely towards each side of the pelvis; but even then we should try, if possible, to seize the child's head by its short diameter. When such a state of things is present, it may be advisable to introduce the posterior blade first, as recommended by Dr. Barnes and many other good authorities. When we recollect how short and inefficient the long forceps used by our grandfathers were, we cannot wonder that they should have felt very shy of performing this high operation; and that when they failed, as they too often did, they felt themselves obliged to have recourse to the horrible alternative of craniotomy. All the best Continental authorities, especially the French, were then much ahead of the British in this respect, simply because they devoted more attention to the subject, and used longer and more efficient instruments.

The number of forceps operations performed by me during the first decade of my practice is sufficient proof of the disinclination to use the forceps (and especially the long forceps), which was then so prevalent. I used the forceps in only seven cases of difficult labour in a total of 308 deliveries between the years 1842 and 1852. The mothers all did well; but one child had been dead for some hours before birth, and another died of pyæmia about a month after. The head was much compressed by the forceps before birth. The forceps used in every case was Denman's short straight one. The blades of this instrument approached too near together, and it had consequently a great compressing power. I am sorry to say that during this decade I had to resort to craniotomy in two cases, which I feel convinced might now have been successfully delivered by the long forceps. In both there was slight narrowing of the conjugate diameter of the pelvic brim, and the head consequently would not enter the pelvic cavity; no ear could be felt after many hours of extreme suffering, and the patients had both become so exhausted that my father, who consulted with me each time, thought that craniotomy was the only alternative. On looking at the kind of long forceps that he then used, which was Ramsbotham's, and which is deficient both in length and in lateral curve, I believe that his judgment was right. During the course of this decade, just before the middle of the present century, two most important discoveries were made which conferred inestimable benefits upon the human race. I mean the discovery of anæsthetics, about the year 1846, by two American dentists, Jackson and Morton, and the discovery of the infectious nature of puerperal fever, in 1847, by Semmelweiss of Vienna.

Sir James Simpson did real good service to obstetric science about this time, by making great improvements in the long and short forceps, and by publishing a series of papers on turning as a substitute for the long forceps. The long forceps he contrived is an especially good one. It is at least an inch longer than Ramsbotham's, measuring 13¾ inches, and combines the excellencies of Levret's lock and Naegele's handles. It is still a good deal used, although most practitioners, like myself, have abandoned it for Dr. Barnes's, which is about an inch longer, and a still better instrument. Simpson's short forceps is only 9¾ inches in length; it is straight, like Denman's, and the blades are similar, but wider apart; it has also shanks between the blades and the lock, and this allows it to be used when the head is high in the pelvic cavity. The handles, however, are very short, but still allow one to use quite a sufficient amount of extractive power. It is a very handy instrument, has been used by me a great many times, and I still continue to use it. If, however, I were limited to the use of only one forceps in every case, I should undoubtedly choose Barnes's, for it can be used both when the head is just entering the upper strait and when it is quite low in the pelvic cavity, although I prefer in the latter case a straight instrument, like Simpson's short forceps.

About the year 1856, I discarded Denman's forceps and adopted Simpson's short forceps, which I found to be much more satisfactory, and safer both for mother and child. Perhaps in consequence of this I was led almost insensibly to use the forceps more frequently in difficult labour, and with results that seemed to me very favourable. I find that in 1856 I used this forceps no less than five times. All these were tedious first labours, for which this kind of short forceps is peculiarly well adapted. My record of the first two decades will show very well how gradually I came to appreciate more and more the safety and efficiency of this instrument. From 1842 to 1852 I only used the forceps seven times; but from 1852 to 1862 I used it 28 times, just four times the number of the previous decade. In these, my first two decades, which I shall have by-and-by to compare with my last two, I recorded, in a total of 672 cases, 35 forceps deliveries. In 29 of these I was called in consultation, whereas in six only was I the original attendant. Twenty-eight were primiparæ, and only seven multiparæ. This shows, what would naturally be expected, that it is in first labours that this forceps is so especially useful. In fact, I employ no other *short* forceps than Simpson's at the present time. I delivered with the long forceps only three times. The instruments used were Dr. Davis's, Dr. Clark's, and Sir

James Simpson's long forceps, and the results were favourable to both mother and child. The last-named forceps I found to be by far the best; but before many years had passed I substituted for it that of Dr. Barnes, which proved to be still better.

Before leaving this decade, it would be a great omission if I neglected to record a most important event which took place on the 16th of December, 1858; viz., the Inaugural Meeting of the Obstetrical Society of London, under the presidency of Dr. Rigby. This was the first beginning of a Society which has since done more than any other in this kingdom for the promotion of obstetrical science. It began well by a valuable paper by my friend Dr. Tyler Smith (who was once a pupil at our Bristol School), in which the author pointed out that too frequent craniotomy had hitherto been the opprobrium of British midwifery; for this operation had been performed in this country "twice as often as in France and four times as often as in Germany;" and to cure this sad state of things he advocated, first and foremost, the more general application of the forceps, especially the long forceps. Dr. Tyler Smith's paper was followed soon afterwards by a very complete and practical one by Mr. P. Harper on "A More Frequent Use of the Forceps as a Means of Lessening both Maternal and Fœtal Mortality." The Society which commenced so auspiciously has since done excellent work in claiming for the forceps its proper place in midwifery, and has consequently effected a great saving of infant life. It has also conferred on the science of gynæcology benefits but little inferior to those I have just mentioned in obstetrics. With regard to the third decade, viz., the ten years from 1862 to 1872, which are intermediate between the first two and the last two decades, there is not much to remark, except that they show a gradually increasing use of the forceps with correspondingly favourable results. There were 72 forceps cases in a total of 495 deliveries. In 45 of these I was the consultant and in 27 the original attendant, thus showing a gradual increase of the latter in comparison with the former class. As usual, first labour cases predominated; for there were 53 of these and 19 multiparous cases. Two mothers died and six children, but all these were consultation cases. One maternal death took place six days after delivery, and was caused by uterine phlebitis; the other happened a few days after delivery, and arose from scarlatina, which also attacked the husband. With regard to the six children, one had been dead for some hours; whilst in the five others death clearly resulted from compression of the head during birth, and in one case evidently from pressure by the forceps on the cord, which happened to be round the neck.

My statistics during the next decade (from 1872 to 1882) show 67 forceps cases instead of 72 as in the previous decade; but as the total number of cases of all kinds is larger, the percentage of forceps cases is consequently smaller from 1872 to 1882. This arises from the circumstance that in this decade the cases in which I was the original attendant greatly outnumbered the consultation cases. For instance, in a total of 609 labours attended by me, there were 67 cases of forceps delivery, of which I was in 49 the original attendant, and in 18 only the consultant. Primiparæ, as usual, predominated, there being 50 of them to 17 multiparæ. There was no maternal death, but 12 infants died, half of them in consultation cases.

During this decade, in the year 1875, I must announce a departure from the old safe rule of Denman in his *Aphorisms*, that "the first stage of labour must be perfectly finished before we think of applying the forceps." This innovation on an old established rule of practice was due to the courage and skill of Dr. Johnston, Master of the Rotunda Hospital, Dublin, and was published by him in 1875, in his annual report of that hospital. In certain cases where the child's head is arrested by slight disproportion at the brim of the pelvis, the membranes will often rupture early, before the os uteri is fully dilated. Under such circumstances, the labour will often be rendered exceedingly tedious, simply because the head will not descend upon the os uteri, so as to complete the dilatation. In such cases, he introduced the forceps within an os uteri that would just admit it, grasped the head and brought it down very gradually, so as to dilate the os and effect delivery. Several cases of this kind were related by him, which were attended with most favourable results both to mother and child. Soon after reading his report, I had an opportunity of testing this new practice myself. I was called in to a similar case of difficulty by another practitioner. The head had long been arrested at the pelvic brim, and the os uteri, as correctly as I could measure it with three fingers, was not more than 3 inches in diameter. I used Simpson's long forceps, and after a good deal of time and patience effected delivery safely, both as regards mother and child. Before three years had passed, I happened to meet with three similar cases, in which the diameters of the os were respectively 3 inches, 2½ inches, and not more than the diameter of a crown piece; and the results were as favourable as in the first instance. In one case I failed to deliver with Simpson's long forceps, but had recourse to Barnes's with perfect success. The same thing happened in the second case, and also in the third, in which I made no further trial of Simpson's instrument. Although it was a better long forceps than most of its predecessors, it is in every way, except perhaps in the handles, inferior to Dr. Barnes's, which I had just then begun to use. As regards the use of the forceps before the os uteri is fully dilated, it is an operation that needs all the cautionary words which Dr. McClintock used when it was first described. It requires great patience, care, and circumspection, and might lead to most serious results in unskilful hands. I would not myself have attempted it if I had only had a dozen or two forceps cases as my previous experience.

No one, in the present day, would venture to deny the great benefits resulting to the mother from a timely resort to the forceps in tedious and difficult labour; but as regards the child the prospect is not altogether so *couleur de rose*. In 1878 I read a paper at the Annual Meeting of the British Medical Association, in Bath, on "The Effects of Forceps Delivery on the Infant;" and I stated that my own experience led me to agree with a conclusion of Dr. Galabin's, which he formed after most careful and patient investigation; viz., that "It has not been shown that the majority, or any considerable proportion, of the stillbirths which now occur in Britain would be preventable by a more timely resort to the forceps." It is true that by a timely use of the long forceps many infants are now saved that would formerly have been destroyed by craniotomy; yet it is no less true that in some other cases the forceps is a source of danger to the child by its increasing the previous pressure on the head during a hard labour; and I gave four cases of my own in which the death of the child was clearly due to a cause which, as far as I know, has not hitherto been mentioned by others: I mean the compression of the umbilical cord by the forceps. The cord of the child, as we all know, is very often round its neck; and in the cases mentioned, I actually observed, when I delivered the head, that a loop of the cord had been and was actually being compressed by the forceps against the angle or ramus of the jaw. Here, then, we have a source of danger to the child, arising simply from the use of the forceps, and which cannot be guarded against by any ordinary amount of skill. I have since met with other similar cases; and as the subject may still be said to be *sub judice*, I hope at some future time to refer to them more in detail.

It only remains for me now to give briefly the statistics of my forceps deliveries during the last decade. From From 1882 to 1892 there were 47 forceps cases in 243 deliveries. The most remarkable feature is that the cases in which I was the original attendant were 32, or nearly double those in which I was consultant, which were only 15. One mother died, but in a case where I was a consultant; and five infants, in four of which cases I was consultant.

I must now give a few figures to show the increased frequency with which the forceps has been used in the last two decades, as compared with the first two. From 1842 to 1862, there were 35 forceps cases in a total of 672 deliveries, *i.e.* nearly 1 in 18; whilst from 1872 to 1892 there were 114 forceps cases in a total of 852 deliveries, or about 1 in $7\frac{1}{2}$. Again, in the first two decades the 35 forceps cases consisted of 28 primiparæ and 7 multiparæ; the number of the former being four times that of the latter. This difference is not so marked in the last two decades, for the 114 forceps cases in this period were made up of 82 primiparæ and 32 multiparæ. These numbers very nearly correspond with those given by Dr. Johnston in his report of the Dublin Rotunda Hospital for 1875, for he remarks: "There were 113 cases where we considered it advisable to deliver with the forceps, and 83 of these were primiparæ, or more than two-thirds." There is another difference between the first two and the last two decades which I must explain presently. From 1842 to 1862, I was the consultant in 28 forceps cases, and the original attendant in 7 only; whereas from 1872 to 1892, I was the consultant in 33 cases only and the original attendant in 81. It will be observed from this that the consulting forceps cases in the last two decades exceeded those of the first by five only; whereas the cases in which I was the original attendant exceeded those of the first two by no less than 74. This proves how thoroughly one's mind had become impressed, in the course of years, with the great advantages of a more frequent use of the forceps. Before saying anything more in praise of the forceps, I wish to speak of one or two things that may be alleged in dispraise of it; and first to call attention to the maternal and infantile mortality. The whole number of forceps cases attended by me in 50 years is 220. This consists of two groups; viz., 114 in which I was the original attendant —in this group no mother died; and a second group of 106 in which I was the consultant—in this there were four deaths of mothers; but, as I said before of consultation cases, I will not be responsible for their issue. The first of these, a primipara, died six days after her confinement, from septicæmia; the second, a multipara, from uterine phlebitis; the third, a primipara, from scarlatina, which also attacked her husband; and the fourth, a primipara, from uræmia, which came on after delivery. When we add these two groups together, we have only four deaths in 224 forceps cases, which is exactly one death in 56 cases; not a bad average when compared with statistics collected from other quarters. In the edition of Dr. Churchill's *Manual* of 1855 he gives statistics of forceps mortality obtained from 34 authorities, both British and foreign, and "the total result is, that in 1,284 forceps cases 76 mothers were lost, or about 1 in 16½; and in 1,262 cases 283 children were born dead, or about 1 in 4¾."[1] Now, these statistics of Dr. Churchill's were collected about 40 years ago, at a time when accoucheurs used the forceps with great reluctance, and put off using it as long as possible; whereas nearly all my cases were attended since that time, when the more frequent use of the forceps had become general, and when it was employed earlier in the labour, and before bad symptoms began to appear. The smaller maternal mortality in the latter cases is thus accounted for. In Dr. Churchill's statistics the infantile mortality is as great as 1 in 4¾. It is, however, as I remarked before, still uncertain whether the forceps effects any great saving of infant life. In my 220 cases 32 children were lost, which is a mortality of 1 in 7; but I have no space now to go into the causes of such mortality. The most usual was pressure, either from the long labour, the use of the forceps, or both. Another objection that has been made to the use of the forceps is that it increases the frequency of perineal laceration. This I emphatically deny. It does not in skilful hands, but it does in the hands of the clumsy and ignorant, and under such circumstances becomes a powerful weapon for evil. I was once called in by a practitioner of this description. I found the perineum torn completely through into the rectum,

[1] CHURCHILL'S *Midwifery*, p. 339.

although the head was still high in the pelvis. He had got hold of the head, as he thought, firmly, and was making strong traction, but in a wrong direction, when the forceps suddenly slipped off the head, and hence the accident. Such an accident may ensue also, if when the head is at the pelvic outlet the operator goes on extracting as if it were at the brim; or if, in a primipara, instead of allowing plenty of time for dilatation, he endeavours to overcome with the forceps the resistance of a rigid perineum. I do not think it advisable, under such circumstance, to remove the forceps, unless it grasps the head awkwardly or obliquely; for just at the last it is very useful sometimes, not for making any traction, but for gently guiding the head forwards beneath the pubic arch. Occipito-posterior presentations are very likely to require the forceps, and also to cause perineal laceration, especially if, as the head descends, the occiput does not come round to the front as it usually does, but, on the contrary, turns backwards into the hollow of the sacrum, and remains in that untoward position until it is expelled. There were as many as 35 occipito-posterior positions in the 220, a number much beyond the usual average of such cases.

I have not kept an exact account of the number of perineal lacerations which occurred in my forceps cases, but I have a strong impression that it is very little above that which often occurs in first labours without the forceps. The worst instance of ruptured perineum which I have met with in any of my cases, with or without the forceps, was one in which the tear went through the fibres of the external sphincter ani, but did not involve the recto-vaginal septum. It was stitched up at the time it happened, and healed well as others did; and I never had a case of laceration so bad that it required a subsequent surgical operation.

And now, having given a sketch of the unfavourable side of the question, which shows that it is not so black as it has been painted, I turn to the favourable side, to do full justice to which would require more vivid colours than I possess. It appears from my statistics of the 220 forceps cases that they consisted of 164 primiparæ and 56 multiparæ; *i.e.* very nearly three times as many first labours as there were of others. This corresponds very closely with the experience of others, and shows that first labours, especially, are those in which the forceps is of such invaluable service. There is no doubt whatever that, as a general rule, first labours last much longer and are attended with an incomparably greater amount of suffering than others, and hence probably has arisen the popular notion that they must be more dangerous. Such a notion may have been well founded formerly before anæsthetics were used, and when the forceps was so very seldom employed, but it is by no means correct in the present day. On the contrary, all my own experience goes to prove that first labours are less dangerous than others. Most accoucheurs of experience will, I think, agree with Dr. Churchill, that a prolonged first stage, which we so often get in primiparæ, is not *per se* dangerous; but few will, I think, be disposed to deny that it may indirectly become a source of danger, and that the pain, the loss of sleep, and want of rest attending a prolonged first stage, which has lasted perhaps for several days, may lead to a flagging of the vital powers in the second: and this is the reason, I think, why we so often notice that the progress of the child's head in first labours becomes more and more slow while traversing the pelvic canal, until at last it becomes stationary as it approaches the pelvic outlet, and the forceps is required to complete the labour. There is no doubt, I think, that fifty or sixty years ago, when obstetric practitioners were so reluctant to use the forceps, this state of things did greatly increase the dangers of first labours. Much more so was this the case towards the close of the last century, when men obeyed more implicitly that dangerous rule of Denman's: "that the head of the child should have rested six hours upon the perineum after the cessation of labour pains before the forceps are used."

But in the present day the more frequent and skilful use of the forceps has quite abolished the dangers of a prolonged second stage thus occasioned, so that we are justified in considering that the second stage of labour is not more dangerous to a primipara than it is to a multipara.

My own statistics give abundant proof that the safety of a primipara is not risked by this modern mode of practice. Although the general symptoms may be quite favourable, I never allow the head to be arrested in the pelvic cavity for more than two hours without applying the forceps. That this practice is justified by the result will be evident when I state that since I began practice, in 1842, I have attended 311 first labours, in which I was the original attendant from the beginning to the end of the labour; that I employed the forceps in 79 of these cases—*i.e.* about one in four—and that there has not been a single maternal death in the 311 cases. The forceps, when used in this way, undoubtedly contributes to the safety of the patient by shortening the labour, and thus saving her not only from a great amount of pain, but what is of much more consequence—a great deal of exhaustion. The first evil, no doubt, can be removed by chloroform, but the latter would be increased by it. But the mere abolition of a great amount of pain by thus shortening the labour is quite enough to justify the use of the forceps. No one who has practised midwifery before the days of chloroform, as I have done, and has witnessed the inexpressible anguish and agony which women had to endure during the prolonged second stage of a first labour (although, perhaps, he had at hand an instrument which he was afraid to use too soon), will ever forget it, or cease to thank God, "who alone can order the unruly wills and affections of sinful men," that He should have so ordered the love of pelf, which was both the mainspring of action and the besetting sin of the Chamberlens, that it should in the end, after many years, confer an inestimable benefit on the human race.

---

Since the earlier part of this paper was printed, I have found that I should have given the number of forceps cases in the last decade as 46 instead of 47. Therefore the cases of the last two decades will be 113 instead of 114. On page 168, line 11, for 33 *read* 34, and for 81 *read* 79.

---

A summary of the forceps cases referred to in the foregoing paper is given in tabular form on the following pages.

### EPITOME OF FORCEPS CASES FROM 1842 TO 1892.

POSITIONS OF HEAD:—

Ordinary—1. Occiput to left acetabulum, OLA
2. Do. right do. ORA

3. Forehead to left acetabulum FLA } Occipito-posterior positions.
4. Do. right do. FRA }

| No. | Date. | *Primipara or Multipara.* | *Original Attendant.* | *Consultant.* | *Position of Head.* | *Forceps used.* | *Result to Mother.* | *Result to Child.* | *Complications, Sequelæ, &c.* |
|---|---|---|---|---|---|---|---|---|---|
| 1 | 1842 | Mult. | Dr. Swayne | Mr. Swayne | OLA | Denman's short | Did well | Lived | None. |
| 2 | 1844 | Prim. | Mr. Croft (pupil) | Dr. Swayne | do. | do. | do. | do. | Child died after the month from pyæmia. |
| 3 | 1846 | do. | Mr. Cleave | do. | do. | do. | do. | Stillborn | Child had been dead some hours. |
| 4 | 1848 | do. | Mr. S. H. Swayne | do. | do. | do. | do. | Lived | None. |
| 5 | 1849 | do. | Dr. Swayne | None | ORA | Davis's short | do. | do. | Phlegmasia dolens, slight attack in both legs. |
| 6 | 1849 | Mult. | Mr. Taylor (pupil) | Mr. Swayne and Dr. Swayne | OLA | Denman's short | do. | do. | Right parietal bone bulged in by pressure. |
| 7 | 1851 | Prim. æt. 40 | Dr. Bartley | Dr. Swayne | ORA | do. | do. | do. | None. |
| 8 | 1853 | Prim. | Mr. Leonard, sen. | do. | OLA | do. | do. | Stillborn | Child was putrid. Some *post-partum* hemorrhage. |
| 9 | 1854 | Mult. | Dr. Swayne | None | do. | do. | do. | Lived | Ergot ineffectual. |
| 10 | 1854 | Prim. æt. 32 | Dr. Lancaster | Dr. Swayne | do. | do. | do. | do. | Slight perineal laceration. |
| 11 | 1855 | Prim. | Mrs. Baily (midwife) | do. | ORA | do. | do. | do. | Simpson's uterine tractor failed. |
| 12 | 1855 | do. | Mr. Cooper (pupil) | do. | FRA | do. | do. | do. | None. |
| 13 | 1855 | do. æt. 41 | Mr. Mayor, sen. | do. | OLA | do. | do. | Died | Child died after two hours from pressure. |
| 14 | 1856 | Prim. | Mr. Biggs (pupil) | do. | ORA | do. | do. | Lived | Ergot given with little effect. |
| 1 | 2 | 3 | 4 | 5 | 6 | 7 | 8 | 9 | 10 |

| 1 | 2 | 3 | 4 | 5 | 6 | 7 | 8 | 9 | 10 |
|---|---|---|---|---|---|---|---|---|---|
| 15 | 1856 | Prim. | Mr. Ashley (pupil) | Dr. Swayne | OLA | Denman's short | Did well | Lived | None. |
| 16 | 1856 | do. | Dr. Brookman | do. | do. | do. | do. | Stillborn | Ergot ineffectual; male child, weight 10 lbs. |
| 17 | 1856 | do. æt. 30 | Dr. Swayne | None | do. | Simpson's short | do. | Died | Rigid os uteri. V.S. to 16 oz. Os lacerated. On third day child died. Blood under dura mater. |
| 18 | 1856 | Prim. æt. 30 | do. | None | do. | do. | do. | do. | Child died from pressure. |
| 19 | 1857 | Mult. | do. | None | do. | do. | do. | Lived | None. |
| 20 | 1857 | do. | Mr. Hodges | Dr. Swayne | do. | do. | do. | do. | None. |
| 21 | 1857 | Prim. | Mr. Board (pupil) | do. | do. | do. | Died | Stillborn | The mother died of puerperal fever after six days. |
| 22 | 1857 | do. | Mr. S. H. Swayne | do. | FLA | do. | Did well | do. | Child died from compression of cord, which was round the neck. |
| 23 | 1858 | do. | Mr. Wilson, sen. | do. | OLA | do. | do. | Lived | An occipital presentation. |
| 24 | 1858 | do. | Mr. Crichton | do. | do. | do. | do. | do. | None. |
| 25 | 1858 | do. | Mr. Macdonald | do. | do. | do. | do. | do. | None. |
| 26 | 1859 | do. æt. 40 | Mr. Sawer | do. | do. | do. | do. | do. | None. |
| 27 | 1859 | Prim. | Mr. Wilson, jun. | do. | do. | Clark's long | do. | do. | Head arrested at brim. |
| 28 | 1859 | Prim. æt. 40 | Dr. Swayne | None | OLA | Simpson's short | do. | do. | None. |
| 29 | 1859 | Prim. | do. | None | FRA | do. | do. | do. | Perineum lacerated as far as sphincter ani. |
| 30 | 1860 | do. | Mr. Macdonald | Dr. Swayne | OLA | do. | do. | do. | None. |
| 31 | 1860 | Mult. | Dr. Swayne | None | FLA | do. | do. | do. | None. |
| 32 | 1860 | Prim. | Mr. Wine (pupil) | Dr. Swayne | OLA | do. | do. | do. | None. |
| 33 | 1860 | Prim. | Mr. Wine (pupil) | Dr. Swayne | ORA | Simpson's short | do. | Lived | *Post-partum* hemorrhage from inertia uteri. |
| 34 | 1860 | do. | Mr. Sawer | Dr. Swayne and Mr. Goodeve | OLA | do. | do. | Stillborn | Perineum lacerated to sphincter ani. |

| No. | Date. | Primipara or Multipara. | Original Attendant. | Consultant. | Position of Head. | Forceps used. | Result to Mother. | Result to Child. | Complications, Sequelæ, &c. |
|---|---|---|---|---|---|---|---|---|---|
| 35 | 1861 | Mult. | Mr. Lansdown | Dr. Swayne | ORA | Ramsbotham's long | Did well | Lived | None. |
| 36 | 1862 | Prim. | Mr. Baretti | do. | OLA | Simpson's short | do. | do. | Slight perineal laceration. |
| 37 | 1862 | do. | Dr. Swayne | None | FRA | do. | do. | do. | None. |
| 38 | 1862 | do. | Mr. Corbould | Dr. Swayne | do. | do. | do. | do. | None. |
| 39 | 1862 | do. | Mr. S. H. Swayne | do. | ORA | do. | do. | do. | None. |
| 40 | 1862 | æt. 38 Prim. | Mr. Macdonald | do. | OLA | do. | do. | do. | Piece of exfoliated bone came away from child's head. |
| 41 | 1862 | do. | Mr. Parker | do. | do. | do. | do. | do. | None. |
| 42 | 1862 | do. | Mr. Macdonald | do. | do. | do. | do. | do. | None. Child weighed 10$\frac{3}{4}$ lbs. |
| 43 | 1862 | do. | Mr. T. E. Clark | do. | do. | do. | do. | do. | Prominent sacrum. Conjugate—3 inches. |
| 44 | 1862 | Mult. | Dr. Swayne | None | do. | do. | do. | do. | None. |
| 45 | 1862 | do. | do. | None | do. | do. | do. | do. | None. |
| 46 | 1862 | Prim. | Mr. Sawer | Dr. Swayne | do. | do. | do. | do. | None. |
| 47 | 1863 | æt. 40 Prim. | Mr. Sheppard | do. | do. | do. | Died | do. | Mother died on the ninth day from uterine phlebitis. |
| 48 | 1863 | æt. 30 Prim. | Dr. Marshall | do. | do. | do. | do. | do. | She died from malignant scarlet fever. |
| 49 | 1863 | do. | Mr. Lang | do. | do. | do. | Did well | do. | Some *post-partum* hemorrhage. |
| 50 | 1863 | do. | do. | do. | do. | do. | do. | do. | Slight perineal laceration. |
| 51 | 1863 | do. | Mr. Hamilton | do. | occipital OLA | do. | do. | do. | None. |
| 52 | 1863 | do. | Mr. Macdonald | do. | do. | do. | do. | do. | None. |
| 1 | 2 | 3 | 4 | 5 | 6 | 7 | 8 | 9 | 10 |

| 1 | 2 | 3 | 4 | 5 | 6 | 7 | 8 | 9 | 10 |
|---|---|---|---|---|---|---|---|---|---|
| 53 | 1863 | Mult. | Mr. Sawer | Dr. Swayne | FLA | Simpson's short | Did well | Lived | None. |
| 54 | 1863 | Prim. æt. 36 | Mr. Sheppard | do. | OLA | do. | do. | Stillborn | Child died from pressure on cord, which was round the neck. |
| 55 | 1863 | Prim. | Mr. Hamilton | do. | OLA | do. | do. | Lived | None. |
| 56 | 1863 | do. | Mr. Macdonald | do. | do. | do. | do. | do. | None. |
| 57 | 1864 | æt. 28 Prim. | Mr. S. H. Swayne | do. | FLA | do. | do. | do. | Some *post-partum* hemorrhage; controlled by ergot and pressure. |
| 58 | 1864 | Mult. | Dr. Swayne | None | ORA | do. | do. | do. | Inertia of uterus. Ergot ineffectual. |
| 59 | 1864 | Prim. | Mr. Macdonald | Dr. Swayne | OLA | do. | do. | do. | Adherent placenta, with hemorrhage. |
| 60 | 1864 | do. | Mr. S. H. Swayne | do. | do. | do. | do. | do. | Slight perineal laceration; two sutures; united well. |
| 61 | 1864 | do. | Mr. Hutchins | do. | FLA | do. | do. | Died | Soon after birth child died from pressure on head. |
| 62 | 1864 | do. | Dr. Swayne | None | OLA | do. | do. | Lived | Pedunculated tumour attached to fundus uteri. |
| 63 | 1864 | do. | Mr. Baretti | Dr. Swayne | do. | do. | do. | Stillborn | Child died from head pressure during labour. |
| 64 | 1864 | do. | Dr. Swayne | None | ORA | do. | do. | Lived | Large cephalhæmatoma of child's head. Cured by puncture. |
| 65 | 1865 | Mult. | do. | None | FRA | do. | do. | do. | None. |
| 66 | 1865 | do. | Mr. Sawer | Dr. Swayne | OLA | do. | do. | do. | None. |
| 67 | 1865 | Prim. | Dr. Swayne | None | do. | do. | do. | do. | Some vesical irritation afterwards. |
| 68 | 1865 | do. | do. | None | do. | do. | do. | do. | None. |
| 69 | 1865 | do. | do. | None | do. | do. | do. | do. | None. |
| 70 | 1865 | do. | do. | None | do. | do. | do. | do. | None. |
| 71 | 1865 | do. | Mr. C. Leonard | Dr. Swayne | do. | do. | do. | Lived | None. |
| 72 | 1865 | do. | Mr. S. H. Swayne | do. | do. (with hand) | do. | do. | do. | None. |

| *No.* | *Date.* | *Primipara or Multipara.* | *Original Attendant.* | *Consultant.* | *Position of Head.* | *Forceps used.* | *Result to Mother.* | *Result to Child.* | *Complications, Sequelæ, &c.* |
|---|---|---|---|---|---|---|---|---|---|
| 73 | 1866 | Prim. | Dr. Swayne | None | OLA | Simpson's short | Did well | Lived | None. |
| 74 | 1867 | do. | Mr. Lodge | Dr. Swayne | do. | do. | do. | Stillborn | Child died apparently from pressure of head. |
| 75 | 1867 | do. | Dr. Swayne | None | do. | do. | do. | Lived | None. |
| 76 | 1867 | Mult. | do. | None | do. | do. | do. | do. | Adherent placenta was peeled off by hand. |
| 77 | 1867 | Prim. æt. 40 | do. | None | do. | do. | do. | do. | None. |
| 78 | 1867 | Prim. | Mr. T. E. Clark | Dr. Swayne | do. | do. | do. | do. | None. |
| 79 | 1867 | Mult. | Mr. Norton | do. | ORA | do. | do. | Stillborn | Ergot given without success. |
| 80 | 1868 | Prim. | Mr. S. H. Swayne | do. | OLA | do. | do. | Lived | None. |
| 81 | 1868 | Mult. | do. | do. | do. | Ramsbotham's Long | do. | do. | None. |
| 82 | 1868 | Mult. | Dr. Swayne | None | OLA | Simpson's short | do. | do. | None. |
| 83 | 1868 | Prim. | do. | None | do. | do. | do. | do. | Perineal laceration as far as sphincter. Two sutures. Healed well. |
| 84 | 1868 | Mult. | Dr. Marshall | Dr. Swayne | ORA | do. | do. | do. | Placenta retained by hour-glass contraction and adhesion. |
| 85 | 1868 | Prim. | Dr. Swayne | None | OLA | do. | do. | do. | Perineum lacerated nearly to sphincter. Two sutures. Healed well. |
| 86 | 1869 | Mult. | Mr. Cross | Dr. Swayne | do. | do. | do. | do. | Ergot ineffectual. |
| 87 | 1869 | Prim. | Dr. Swayne | None | do. | do. | do. | do. | None. |
| 88 | 1869 | do. | Mr. T. E. Clark | Dr. Swayne | do. | do. | do. | do. | None. |
| 89 | 1869 | Mult. | Mr. Colthurst | do. | FRA | do. | do. | do. | Occiput turned to the front during extraction. |
| 90 | 1869 | Prim. | Dr. Swayne | None | ORA | do. | do. | do. | Perineum slightly lacerated. Healed well. |
| 1 | 2 | 3 | 4 | 5 | 6 | 7 | 8 | 9 | 10 |

| 1 | 2 | 3 | 4 | 5 | 6 | 7 | 8 | 9 | 10 |
|---|---|---|---|---|---|---|---|---|---|
| 91 | 1869 | Prim. | Mr. S. H. Swayne | Dr. Swayne | ORA | Simpson's short | Did well | Lived | Perineal laceration nearly to sphincter. Three sutures. Healed well. |
| 92 | 1869 | do. | Dr. Swayne | None | do. | do. | do. | do. | None. |
| 93 | 1869 | do. | Mr. Rossiter | Dr. Swayne | FRA | do. | do. | Stillborn | Died from head pressure. Arm wanting. |
| 94 | 1869 | do. | Dr. Swayne | None | ORA | do. | do. | Lived | Placenta retained by hour-glass contraction. |
| 95 | 1869 | do. | Mr. Coe | Dr. Swayne | do. | do. | do. | do. | Perineum slightly lacerated. Sutures. Healed well. |
| 96 | 1870 | do. | Mr. S. H. Swayne | do. | FRA | do. | do. | do. | None. |
| 97 | 1870 | Mult. | Dr. Swayne | None | ORA | do. | do. | do. | Hour-glass contraction. Placenta wholly adherent. |
| 98 | 1870 | do. | do. | None | do. | do. | do. | do. | Placental adhesion and hemorrhage. |
| 99 | 1870 | Prim. | do. | None | FLA | do. | do. | do. | None. |
| 100 | 1870 | Mult. | do. | None | OLA | do. | do. | do. | None. |
| 101 | 1871 | do. | Mr. Cross | Dr. Swayne | FRA | do. | do. | do. | None. |
| 102 | 1871 | do. | Dr. Swayne | None | ORA | do. | do. | { do. Died } | Twins. Second child died a few hours after birth; it was expelled naturally. |
| 103 | 1871 | do. | Mr. Smith | Dr. Swayne | do. | Ramsbotham's long | do. | Lived | Patient had prominent sacrum. |
| 104 | 1871 | Prim. | Dr. Swayne | None | do. | Simpson's short | do. | do. | Forceps pressure produce temporary paralysis of face. |
| 105 | 1871 | do. | Mr. Mayor | Dr. Swayne | do. | do. | do. | do. | Some hemorrhage *post partum*; restrained by pressure and ergot. |
| 106 | 1871 | do. | Mr. Ring | do. | do. | do. | do. | do. | None. |
| 107 | 1871 | do. | Mr. C. Reade | do. | do. | do. | do. | do. | None. |
| 108 | 1872 | Mult. | Dr. Swayne | None | OLA | do. | do. | do. | None. |
| 109 | 1872 | Mult. | Mr. Ellis | Dr. Swayne | ORA | Barnes's | do. | Lived | None. |
| 110 | 1872 | Prim. | Dr. Swayne | Mr. S. H. Swayne | FLA | do. | do. | Dead | Rigid os. Narrow pelvis. Forehead of child indented by forceps. Adherent placenta. |

| *No.* | *Date.* | *Primipara or Multipara.* | *Original Attendant.* | *Consultant.* | *Position of Head.* | *Forceps used.* | *Result to Mother.* | *Result to Child.* | *Complications, Sequelæ, &c.* |
|---|---|---|---|---|---|---|---|---|---|
| 111 | 1872 | Mult. | Dr. Swayne | None | FRA | Simpson's short | Did well | Lived | Hour-glass contraction and adhesion of placenta. |
| 112 | 1872 | do. | Mr. Corbould | Dr. Swayne | OLA | Barnes's | do. | Stillborn | Child died from pressure on head. |
| 113 | 1872 | do. | Dr. Swayne | None | do. | do. | do. | Lived | Membranes punctured for hydramnios. |
| 114 | 1872 | Prim. | Mr. Corbould | Dr. Swayne | ORA | Simpson's short | do. | do. | She had pleurisy during her recovery. |
| 115 | 1873 | do. | Dr. Swayne | None | FLA | do. | do. | do. | None. |
| 116 | 1873 | do. | do. | None | OLA | do. | do. | do. | Perineum torn nearly to sphincter. One stitch. Healed well. |
| 117 | 1873 | do. | do. | None | do. | do. | do. | do. | Perineum torn to sphincter. Two stitches. Healed well. |
| 118 | 1873 | do. | do. | None | do. | do. | do. | do. do. } | Twins. First, breech. Second, head—forceps. |
| 119 | 1873 | Mult. | do. | None | ORA | Barnes's | do. | do. | None. |
| 120 | 1873 | Prim. | do. | None | do. | Simpson's short. | do. | do. | None. |
| 121 | 1873 | Mult. | Mr. Talbot | Dr. Swayne | do. | Simpson's long | do. | Stillborn | Cord round the neck compressed by forceps. |
| 122 | 1873 | Prim. | Dr. Swayne | None | do. | Simpson's short | do. | Lived | *Post-partum* hemorrhage stopped by ergot, turpentine and pressure. |
| 123 | 1874 | do. æt. 34. | Mr. S. H. Swayne | Dr. Swayne | FLA | do. | do. | do. | Hour-glass contraction and adhesion of placenta. |
| 124 | 1874 | Prim. | Dr. Swayne | None | OLA | do. | do. | do. | Perineum lacerated to sphincter. Two sutures. Healed well. |
| 125 | 1874 | do. | do. | None | FRA | do. | do. | do. | Slight perineal laceration. One suture. Healed well. |
| 126 | 1874 | do. | do. | None | OLA | do. | do. | do. | None. |
| 127 | 1874 | do. | do. | None | do. | do. | do. | do. | Perineum lacerated. Two sutures. Healed well. |
| 128 | 1875 | do. | do. | None | ORA | do. | do. | do. | Partial inversio uteri. Easily reduced. |
| 1 | 2 | 3 | 4 | 5 | 6 | 7 | 8 | 9 | 10 |

| 1 | 2 | 3 | 4 | 5 | 6 | 7 | 8 | 9 | 10 |
|---|---|---|---|---|---|---|---|---|---|
| 129 | 1875 | Prim. | Mr. Parker | Dr. Swayne | OLA | Simpson's short | Did well | Lived | Placenta retained by irregular contraction. |
| 130 | 1875 | Mult. | Dr. Swayne | None | FRA | do. | do. | do. | None. |
| 131 | 1875 | do. | do. | None | ORA | do. | do. | do. | Hour-glass contraction. Adherent placenta. |
| 132 | 1875 | Prim. | Mr. Ruddock | Dr. Swayne | OLA | do. | do. | do. | Laceration of perineum. Two sutures. Healed well. |
| 133 | 1875 | do. | Mr. James | None | do. | Simpson's long | do. | do. | None. |
| 134 | 1875 | Mult. | Dr. Swayne | None | do. | Barnes's | do. | do. | None. |
| 135 | 1876 | Prim. | do. | None | do. | Simpson's short | do. | do. | None. |
| 136 | 1876 | do. | do. | None | ORA | do. | do. | do. | Perineum torn through external sphincter. Four sutures. Healed well. |
| 137 | 1876 | Prim. æt. 36. | Dr. Swayne | None | OLA | do. | do. | do. | Perineum lacerated to sphincter. Two sutures. Healed well. |
| 138 | 1876 | Prim. | do. | None | FRA | Barnes's | do. | do. | Os uteri size of crown piece when forceps was used. Lacerated perineum. Healed well. |
| 139 | 1876 | do. æt. 36 | do. | None | do. | do. | do. | Stillborn | Child appeared to have been dead some hours, as skin had peeled. |
| 140 | 1876 | Prim. | do. | None | FLA | do. | do. | Lived | Perineum torn to sphincter. Two sutures. Healed well. |
| 141 | 1876 | Mult. | Mr. Mayor | Dr. Swayne | ORA | do. | do. | do. | None. |
| 142 | 1877 | Prim. | Dr. Swayne | None | OLA | Simpson's short | do. | do. | Perineum lacerated to sphincter. Two sutures. Healed well. |
| 143 | 1877 | do. | do. | None | ORA | Barnes's | do. | do. | Phlegmasia dolens a fortnight after delivery. Good recovery. |
| 144 | 1877 | do. æt. 37 | do. | None | FLA | Simpson's short | do. | do. | Adherent placenta. Removed by hand. |
| 145 | 1877 | Mult. | do. | None | OLA | Barnes's | do. | Stillborn | Very prominent sacrum. Child had been dead some hours. |
| 146 | 1877 | do. | do. | None | FLA | do. | do. | Lived | None. |
| 147 | 1877 | do. | Dr. Imlay | Dr. Swayne | OLA | Barnes's | do. | Stillborn | Child died from compression of slightly prolapsed cord against forehead. |
| 148 | 1887 | do. | Dr. Lawrence | do. | do. | do. | do. | Lived | Adherent placenta removed by hand. |

| *No.* | *Date.* | *Primipara or Multipara.* | *Original Attendant.* | *Consultant.* | *Position of Head.* | *Forceps used.* | *Result to Mother.* | *Result to Child.* | *Complications, Sequelæ, &c.* |
|---|---|---|---|---|---|---|---|---|---|
| 149 | 1878 | Prim. | Dr. Swayne | None | ORA | Simpson's short | Did well. | Died | Child died convulsed on third day. Large cephalhæmatoma from pressure. |
| 150 | 1878 | do. | do. | None | OLA | do. | do. | Lived | Slight perineal laceration; did not unite owing to cough. |
| 151 | 1878 | Mult. | do. | None | FLA | Barnes's | do. | do. | Ergot ineffectual. |
| 152 | 1878 | Prim. | Dr. Challacombe | Dr. Swayne | OLA | do. | do. | do. | None. |
| 153 | 1878 | do. | Dr. Swayne | None | do. | Simpson's short | do. | Died | Child died from pressure. *Post-partum* hemorrhage restrained by ice and pressure. |
| 154 | 1878 | æt. 45 Prim. | do. | None | do. | do. | do. | Lived | Child had sloughing ulcer on parietal eminence, and crack behind the ear. |
| 155 | 1878 | do. | do. | None | ORA | Barnes's | do. | do. | Slight laceration of perineum. One suture. Healed well. |
| 156 | 1878 | do. | do. | None | OLA | do. | do. | do. | Phlegmasia dolens of both legs a fortnight after delivery. |
| 157 | 1879 | æt. 40 Mult. | do. | None | do. | do. | do. | do. | None. |
| 158 | 1879 | Prim. | Mr. S. H. Swayne | Dr. Swayne | do. | do. | do. | do. | None. |
| 159 | 1879 | do. | Dr. Swayne | None | do. | Simpson's short | do. | do. | None. |
| 160 | 1879 | Mult. | Mr. S. H. Swayne | Dr. Swayne | do. | Barnes's | do. | do.<br>do. } | Twins. First delivered by forceps; second by nature. Retained placenta and hemorrhage. |
| 161 | 1879 | Prim. | Dr. Swayne | None | do. | do. | do. | do. | None. |
| 162 | 1880 | do. | Mr. S. H. Swayne | Dr. Swayne | FRA | Denman's short | do. | do. | Perineum torn to sphincter ani. Two sutures. Good union. |
| 163 | 1880 | do. | Mr. Mayor | do. | OLA | Barnes's | do. | Stillborn | Child appeared to have been dead some hours. Perineal laceration. Healed well. |
| 164 | 1880 | æt. 30 Prim. | Dr. Challacombe | do. | do. | do. | do. | Lived | None. |
| 165 | 1881 | Prim. | Dr. Swayne | None | OLA | Simpson's short | do. | do. | Perineum torn to sphincter. Two sutures. Healed well. |
| 166 | 1881 | do. | do. | None | do. | Barnes's | do. | do. | Do. Do. |
| 1 | 2 | 3 | 4 | 5 | 6 | 7 | 8 | 9 | 10 |

| 1 | 2 | 3 | 4 | 5 | 6 | 7 | 8 | 9 | 10 |
|---|---|---|---|---|---|---|---|---|---|
| 167 | 1881 | Prim. | Dr. Swayne | None | ORA | Barnes's | Did well | Lived | None. |
| 168 | 1881 | Mult. | Mr. Baretti | Dr. Swayne | FLA | do. | do. | Stillborn | Child apparently died from long pressure. |
| 169 | 1881 | Prim. | Mr. Dobson | do. | OLA | do. | do. | do. | Patient had dysuria and bloody urine for some days. |
| 170 | 1881 | Mult. | Dr. Swayne | None | do. | do. | do. | Lived | None. |
| 171 | 1881 | Prim. | do. | None | FLA | do. | do. | do. | None. |
| 172 | 1881 | do. | do. | None | OLA | Simpson's short | do. | do. | Perineum torn to sphincter. Two sutures. Healed well. |
| 173 | 1881 | do. | do. | None | ORA | Barnes's | do. | do. | Do. Do. |
| 174 | 1881 | do. | do. | None | FRA | do. | do. | Died | Child died in an hour from large exomphalos and ectopia cordis. |
| 175 | 1882 | do. | do. | None | ORA | do. | do. | Lived | None. |
| 176 | 1882 | do. | Mr. S. H. Swayne | Dr. Swayne | FLA | do. | do. | do. | Paralysis of left side of child's face from forceps pressure. |
| 177 | 1882 | do. | Mr. Lansdown | do. | ORA | do. | Died | do. | She died after five days from uræmia and albuminuria. Child had a slough behind each ear. |
| 178 | 1882 | do. | Dr. Swayne | None | OLA | do. | Did well | do. | Lacerated perineum. Two sutures. Healed well. |
| 179 | 1882 | Mult. | do. | None | do. | do. | do. | do. | None. |
| 180 | 1882 | do. | do. | None | do. | Simpson's short | do. | do. | None. |
| 181 | 1883 | Prim. | do. | None | do. | do. | do. | do. | None. |
| 182 | 1883 | Mult. | do. | None | do. | do. | do. | Stillborn | Child appeared to have been dead for many days. Hydramnios. Labour induced. |
| 183 | 1883 | do. | do. | None | do. | do. | do. | Lived | Want of uterine power. Ergot given with good effect. |
| 184 | 1883 | Prim. | do. | None | do. | do. | do. | do. | Hour-glass contraction, and adherent placenta. |
| 185 | 1884 | Prim. | do. | None | do. | do. | do. | do. | Slight laceration of perineum. One suture. Healed. |
| 186 | 1884 | do. | do. | None | do. | do. | do. | do. | None. |

| *No.* | *Date.* | *Primipara or Multipara.* | *Original Attendant.* | *Consultant.* | *Position of Head.* | *Forceps used.* | *Result to Mother.* | *Result to Child.* | *Complications, Sequelæ, &c.* |
|---|---|---|---|---|---|---|---|---|---|
| 187 | 1884 | Mult. | Dr. Swayne | None | OLA | Simpson's short | Did well | Lived | None. |
| 188 | 1884 | Prim. | do. | None | do. | do. | do. | do. | None. |
| 189 | 1884 | do. æt. 33 | do. | None | ORA | Barnes's | do. | do. | Slight perineal tear. One suture. Healed well. |
| 190 | 1884 | Prim. | do. | None | OLA | Simpson's short | do. | do. | Do. do. |
| 191 | 1884 | Mult. | do. | None | do. | Barnes's | do. | do. | None. |
| 192 | 1885 | Prim. | do. | None | do. | Simpson's short | do. | do. | Perineum torn to sphincter. Two sutures. Healed well. |
| 193 | 1885 | do. | do. | None | FRA | Barnes's | do. | do. | Perineum torn to sphincter. Two sutures. Healed well. |
| 194 | 1885 | do. | Mr. S. H. Swayne | Dr. Swayne | OLA | do. | do. | do. | *Post-partum* hemorrhage, checked by ergot and pressure. |
| 195 | 1885 | Mult. | Dr. Swayne | None | ORA | do. | do. | do. | None. |
| 196 | 1885 | do. | Mr. Baretti | Dr. Swayne | do. | do. | do. | do. | None. |
| 197 | 1886 | Prim. æt. 40 | Dr. Swayne | None | OLA | do. | do. | Stillborn | None. |
| 198 | 1886 | Prim. | do. | None | ORA | do. | do. | Lived | Perineum ruptured to sphincter ani. Two sutures. Healed well. |
| 199 | 1887 | do. | do. | None | do. | Simpson's short | do. | do. | Perineal tear went through external sphincter. Two sutures. Healed well. |
| 200 | 1887 | do. æt. 35 | do. | None | OLA | Barnes's | do. | do. | None. |
| 201 | 1887 | Prim. æt. 36 | do. | None | ORA | Simpson's short | do. | do. | Perineum torn to sphincter. Three sutures. Healed well. Hour-glass contraction. |
| 202 | 1887 | Mult. | Mr. Corbould | Dr. Swayne | FRA | Barnes' | do. | do. | None. |
| 203 | 1888 | Prim. | Dr. Swayne | None | OLA | Simpson's short | do. | Stillborn | Child died from pressure. Lacerated perineum. One suture. Healed well. Hour-glass contraction. Hand used. |
| 204 | 1888 | do. | do. | None | ORA | do. | do. | Lived | Hour-glass contraction. |
| 1 | 2 | 3 | 4 | 5 | 6 | 7 | 8 | 9 | 10 |

| 1 | 2 | 3 | 4 | 5 | 6 | 7 | 8 | 9 | 10 |
|---|---|---|---|---|---|---|---|---|---|
| 205 | 1888 | Mult. | Dr. Swayne | None | FRA | Simpson's short | Did well | Lived | *Post-partum* hemorrhage, checked by pressure and ergot. |
| 206 | 1888 | Prim. | Mr. S. H. Swayne | Dr. Swayne | OLA | Barnes's | do. | do. | Partial laceration of perineum. Two sutures. Healed well. |
| 207 | 1888 | Mult. | Dr. Swayne | None | FLA | do. | do. | do. | Placenta retained by hour-glass contraction. |
| 208 | 1889 | Prim. | do. | None | OLA | do. | do. | do. | Perineal tear to sphincter. Two sutures. Healed well. |
| 209 | 1889 | Mult. | Mr. Griffiths | Dr. Swayne | FLA | Simpson's short | do. | do. | None. |
| 210 | 1889 | Prim. æt. 38 | Dr. Lees | do. | do. | do. | do. | do. | Perineal laceration to sphincter. Two sutures. Healed well. |
| 211 | 1889 | Mult. | Dr. Swayne | None | do. | do. | do. | do. | Some hour-glass contraction of uterus. |
| 212 | 1890 | Prim. | Mr. Fendick | Dr. Swayne | do. | Barnes's | do. | Stillborn | Bad laceration of perineum. Union not complete. No after-operation. |
| 213 | 1890 | do. | Dr. Swayne | None | ORA | Simpson's short | do. | Lived | None. |
| 214 | 1890 | do. | Mr. Baretti | Dr. Swayne | OLA | do. | do. | Stillborn | Child died from pressure. Some hour-glass contraction. |
| 215 | 1890 | do. | Dr. Swayne | None | do. | do. | do. | Lived | Slight perineal tear. One suture. Healed well. |
| 216 | 1890 | do. | do. | None | do. | do. | do. | do. | Hemorrhage from irregular contraction. Hand used. |
| 217 | 1891 | Mult. | Mr. Baretti | Dr. Swayne | do. | Barnes's | do. | do. | Child marked on forehead each side. |
| 218 | 1891 | Prim. | Dr. Colman | do. | do. | do. | do. | do. | Perineal laceration. Two sutures. Healed well. |
| 219 | 1891 | do. | Mr. M. Smith | do. | FLA | Simpson's short | do. | Stillborn | Child died from pressure on cord by forceps. |
| 220 | 1891 | do. | Mr. Ewens | do. | FRA | Simpson's long | do. | do. | Child died from pressure on head or cord. |

